# Measurement and Evaluation of Health, Functional Capacity, Physical Fitness, and Daily Habits of Greek Female Healthcare Professionals Working in a Hospital Environment

**DOI:** 10.3390/healthcare13040383

**Published:** 2025-02-11

**Authors:** Anastasia Chasandra, Konstantina Karatrantou, Kyriaki Papazeti, Anastasia Melissopoulou, Christos Batatolis, Maria Mourounoglou, Rafaela-Varvara Sioupi, Vassilis Gerodimos

**Affiliations:** 1 Department of Physical Education and Sports Science, University of Thessaly, 42100 Trikala, Greece; 2 Pella General Hospital-Edessa Hospital Unit, 58200 Edessa, Greece; 3 General Hospital of Volos “Achilopoulion”, 38222 Volos, Greece; 4 Pella General Hospital-Giannitsa Hospital Unit, 58100 Giannitsa, Greece; 5 Faculty of Medicine, Medical University of Sofia, 1463 Sofia, Bulgaria

**Keywords:** wellness, lifestyle, musculoskeletal pains, physical activity, unhealthy behaviors, workplace

## Abstract

Background/Objectives: The wellness profile of healthcare professionals is of crucial importance since it can affect the quality of healthcare services. This study created a comprehensive profile of health, functional capacity, physical fitness, and lifestyle behaviours in hospital healthcare professionals. Methods: A hundred female (age: 45.53 ± 2 yrs) professionals underwent assessments of their health (respiratory function/blood pressure/body circumference/body fat/body mass index/musculoskeletal pains), functional capacity (flexibility/balance), physical fitness (strength/aerobic capacity), and daily habits (smoking/alcohol/caffeinated beverages/screen time/physical activity). Results: According to data analysis (descriptive statistics, paired *t*-tests to analyze possible differences between the right and left sides), (a) 39% of the participants were overweight and 28% obese, (b) 40–44% of the participants showed higher values than normal in waist circumference and systolic blood pressure, and (c) 96% of the participants showed musculoskeletal pains. Functional capacity and physical fitness demonstrated significant differences in balance and flexibility (*p* < 0.05) between sides (right/left) and low levels of strength and dynamic balance-agility. Moreover, 67% of the sample did not participate in physical activities, while a significant percentage showed increased screen time on weekdays (44%) and weekends (61%). Finally, 36% of the sample smoked, 62% consumed alcohol, and 92% consumed caffeinated beverages (the majority were within permissible limits). Conclusions: In conclusion, this study contributes valuable insights into the wellness profile of healthcare professionals to ensure optimal care for this population.

## 1. Introduction

Health workers represent a significant proportion of the total working population worldwide (65.1 million health workers in 2020, while the projected health workforce size by 2030 is 84 million health workers) [1] and play an essential role in detecting disease, caring for the sick and wounded, and engaging with affected communities to educate and influence people’s health behaviors [2]. Theoretically, health workers should have the necessary knowledge to adopt a healthy lifestyle and be a core group in the general population’s health promotion strategies (i.e., encouraging daily physical activity) [3]. Unfortunately, health workers’ knowledge about healthy lifestyle behaviors does not always translate into healthier choices in their daily life, as they present increased morbidity (i.e., obesity, metabolic syndrome, hypertension) compared to the general population, showing that they are not taking good care of their health [4,5]. For this reason, during recent decades, researchers from different countries all over the world designed and implemented workplace wellness interventions (regarding physical activity and exercise, nutrition, mental health, ergonomics, etc.) to improve various indices of physical (body composition, blood pressure, respiratory function, musculoskeletal pains) and mental health, functional capacity (flexibility, balance), physical fitness (strength, aerobic capacity), quality of life, and lifestyle behaviors (eating habits, smoking, sleeping, alcohol, physical activity, and sedentary habits) in healthcare workers [6,7,8,9,10].

According to the Centers for Disease Control and Prevention (CDC) ‘Workplace Health Model’ [11], a successful workplace health program targets the specific working population and fits the workplace, workers’ needs, as well as personal and organizational health goals. This is achieved through a specialized initial health assessment [11]. The initial health and wellness screening of healthcare workers (using various tests and/or questionnaires for the assessment of health parameters, functional capacity/physical fitness indices, and lifestyle behaviors), in conjunction with the creation of reference values/ranking tables for the specific working population and the detection of problems/needs, will provide valuable information for the effective design and implementation of workplace wellness interventions, aiming to improve workers’ health and quality of life as well as working ability and productivity [12,13].

In the scientific literature, there are several studies on different specialties of health workers all over the world (including Greece) that mainly focus on the evaluation of quality of life and mental health parameters (i.e., anxiety, distress, depression, satisfaction, burnout) [14,15,16,17,18,19,20,21,22,23,24,25,26]. Previous studies examined indicators of physical health involving mainly body composition, blood pressure, and/or musculoskeletal pain assessment (some of them focused on isolated or selected body parts such as the neck, lumbar spine, upper back, and/or shoulder) [27,28,29,30,31,32,33,34,35], while the information regarding the comprehensive musculoskeletal pain profile of the total body, the functional capacity, and physical fitness levels is more limited for healthcare workers. However, the evaluation of functional capacity and physical fitness levels is of crucial importance, since previous studies have demonstrated that healthcare workers should have adequate levels of flexibility, strength, and aerobic capacity to cope with the different physical demands of their job (i.e., continuous walking, lifting and transferring patients, quickly move during an emergency) [36]. Furthermore, a previous study by Hansen et al. [37] demonstrated a significant association between aerobic capacity and maximal isometric elbow strength with the quality of cardiocerebral resuscitation in healthcare professionals. Additionally, although, lifestyle behaviors may positively or negatively affect different health parameters, most previous studies all over the world mainly focused on the evaluation of smoking, alcohol consumption, and sleep [38,39,40,41,42,43,44], whereas the information regarding caffeinated beverage consumption, sedentary behaviors, and physical activity levels is limited in the health worker population [45,46,47].

It should be also mentioned that in some countries (i.e., Greece), there is a great lack of information regarding the physical activity and sedentary habits as well as the functional capacity and physical fitness levels of health workers compared to other countries. Taking all the above into consideration, the measurement and the evaluation of a comprehensive wellness profile specifically for the Greek health worker population, where the work conditions, work demands, structure and function of healthcare system, and musculoskeletal complaints differ compared to other countries [28,35], is of crucial importance. For example, a previous study that compared musculoskeletal disorders between Greek and Dutch nursing personnel demonstrated that Greek nurses reported significantly more back and neck complaints in the preceding 12 months than the Dutch workers [28].

Thus, the purpose of this study was to measure, evaluate, and create a wide-ranging wellness profile of health indices (body composition and circumference, blood pressure, respiratory function, musculoskeletal pains in nine body parts), functional capacity (flexibility of lower and upper limbs, static and dynamic balance), physical fitness (strength and aerobic capacity), and lifestyle behaviors (smoking, alcohol, and caffeinated beverage consumption, screen time levels, and physical activity levels) for Greek female healthcare professionals working in public hospital environments.

## 2. Materials and Methods

### 2.1. Participants

One hundred female healthcare professionals of different specialties (e.g., doctors, nurses, ergotherapists, physiotherapists, medical laboratory technicians) from two public hospitals in Greece (General Hospital of Volos “Achilopoulion” and General Hospital of Edessa), volunteered to participate in the present study (Table 1). To take part in the present study, the participants should be (a) females, (b) health professionals who worked at public hospitals, and (c) under 60 years old. Furthermore, the participants should have a permanent position in the hospital, with at least two years of previous working experience. Male participants, as well as participants above 60 years old or participants who worked as administrative staff for the hospital, were excluded from the present study. Before the start of the study, participants’ health status was assessed by a standardized health history questionnaire as per the American College of Sports Medicine’s guidelines [48]. The research participants were informed in detail about the measurement procedures and signed, after studying it carefully, a consent form. The study was initially approved by the Institutional Ethics Committee of the University of Thessaly, then by the hospital’s Scientific Council, as well as by the Administration of the 3rd Health Region of Macedonia. It should be also mentioned that all procedures followed the Helsinki Declaration.

### 2.2. Study Design—Procedure

The study employed a cross-sectional design. All measurements were performed by the investigator out of the workplace on participants’ day off, since they were more rested. The participants came to the assessment area at a predetermined time (09:00 a.m.–12:00 p.m.). Initially, they filled in the questionnaires for the evaluation of musculoskeletal pains and lifestyle behaviors. Thereafter, the measurements of health (anthropometric characteristics, body mass index, body fat and circumference, blood pressure, respiratory function), functional capacity (flexibility, balance), and physical fitness (strength, aerobic capacity) were performed, following the aforementioned order. Before the functional capacity and physical fitness measurements, all the participants performed a standardized 12 min warm-up, including 5 min stationary cycling and 7 min static and dynamic stretching exercises for the total body. Following measurements, the participants performed a cool-down consisting of 3 min low-intensity stationary cycling and 5 min static stretching exercises. Before testing, the participants were instructed to (a) abstain from any caffeine, tobacco, and alcohol consumption for at least 24 h, (b) avoid intense physical activity for the last 48 h, (c) follow their normal diet, and (d) have sufficient sleep the previous night.

### 2.3. Measurements

During the study, selected health, functional capacity, and physical fitness indices as well as lifestyle behaviors were assessed (Table 2) using various internationally recognized and widely used tests and questionnaires. The test-retest reliability of the functional capacity and physical fitness tests has been previously examined in other studies [49,50,51,52], reporting good/high test-retest reliability (ICC = 0.85–0.98) in middle-aged and older individuals.

### 2.4. Statistical Analysis

All statistical analyses were performed using IBM SPSS Statistics v.26 software (IBM Corporation, Armonk, New York, NY, USA). A statistical power analysis (software package GPower 3.0) before the initiation of the study indicated that a total number of 100 participants would yield adequate power (>0.85) and a level of significance (<0.05). The normality of data distribution for the total sample was checked using the Kolmogorov–Smirnov test and all variables were found to follow a normal distribution (Supplementary Table S1), while skewness and kurtosis values were also presented (Supplementary Table S1). Mean, standard deviation, median, minimum, and maximum values, as well as frequency tables, were used to analyze the data and to create a comprehensive wellness profile. Furthermore, paired *t*-tests were also used to analyze possible differences between the right and left sides in the back scratch and single-limb stance tests. The level of significance for all statistical analyses was set at *p* < 0.05.

## 3. Results

Initially the results of the present study were examined separately between the two hospitals. However, no significant differences (*p* > 0.05) were observed and for this reason in our manuscript we present the results of all the participants as a total sample.

### 3.1. Health Indices

#### 3.1.1. Musculoskeletal Pains

**Figure 1:**
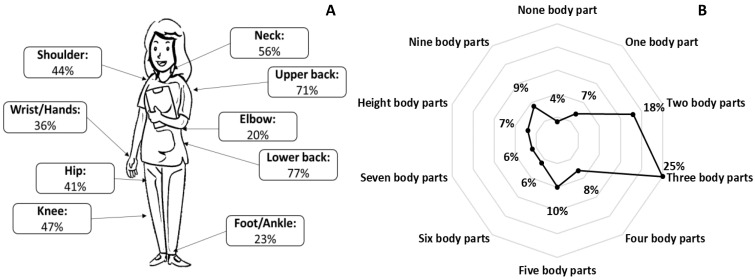
Incidence of musculoskeletal pains in different body parts (**A**) and number of pained body parts (**B**).

The results of the study showed that 96% of healthcare professionals had musculoskeletal pains in different body parts (Figure 1A) and only 4% had no musculoskeletal pains. The three body parts with the highest incidence of pain were the lower back (77%), upper back (71%), and neck (56%). It should be also mentioned that the vast majority of healthcare professionals (89%) had musculoskeletal pains in more than one body part (Figure 1B). The majority of healthcare professionals (94%) were not absent from work at all; however, a great proportion of them (58%) had difficulties performing their daily activities at work due to musculoskeletal pains. Furthermore, a significant proportion of the sample (32%) stated that these musculoskeletal pains hindered their daily activities for more than 10 days, whereas 14% of the sample stated that every day they had difficulties in their daily activities due to musculoskeletal pains.

Regarding the duration of pain, the vast majority of the participants reported that their pains in different body parts (shoulder, hip, and knee: 24–25%; elbow, wrist, and foot: 11–19%; neck, upper back, and lower back: 31–43%) lasted for 1–7 days during the preceding month. However, a significant proportion of the participants stated that their pains lasted from more than 22 days to every day during the last month in the hip or knee (10–11%), shoulder and neck (13–15%), and in the upper back and lower back (23–26%). The duration of pain in the nine body parts is analytically presented in Table 3.

The intensity of pain on a 10-point scale was either 3–5 or ≥6 for the majority of the pained participants in the neck (26% and 22%, respectively), upper back (31% and 30%, respectively), lower back (26% and 38%, respectively), and knee (21% and 16%, respectively) body parts. In body parts such as the elbow, wrist/hands, and foot/ankle, the intensity of pain for the vast majority of the pained participants ranged from 1 to 2 in a 10-point scale. The intensity of pain in the nine body parts is analytically presented in Table 4.

#### 3.1.2. Other Health Indices

The results of the study showed that 33% of the healthcare professionals had a normal body mass index (BMI) (18.5–24.9% kg/m^2^), 39% were overweight (above 25 and below 30 kg/m^2^), and 28% were obese (≥30 kg/m^2^), according to the ACSM classification [48]. The average BMI of the participants was approximately 27.37, indicating that on average individuals fell within the overweight category. However, the group’s BMI ranged from 18.5 to 37.96, suggesting a diverse range of body composition within the sample. The mean body fat percentage is approximately 35.92%, with a great variability ranging from 18.4% to 48.90%. The mean systolic blood pressure was 122.6 mmHg, and the mean diastolic blood pressure was 75.6 mmHg. These values are within the normal range, but the standard deviations indicate some variability among participants, especially when 25% of participants recorded having systolic blood pressure of more than 130 mmHg, and diastolic blood pressure was ≥85 mmHg for 20% of the participants, which is considered as pro-hypertension or the hypertension stage, according to the ACSM classification [48]. Concerning the spirometry measurements, while the mean values appeared within typical ranges, the wide range of observed values suggests variability in respiratory health among participants. Finally, the mean waist and hip circumference values were 88.3 cm and 101.7 cm, respectively, with considerable variability observed across participants. It should also be mentioned that a significant proportion of the participants (45–49%) demonstrated a waist circumference of ≥88 cm and a waist-to-hip ratio of ≥0.86, indicating, therefore, high risk for the presence of cardiovascular diseases, according to the ACSM classification [48]. The descriptive statistics of body composition and circumference, blood pressure, and respiratory function are analytically presented in Table 5.

### 3.2. Functional Capacity and Physical Fitness

#### 3.2.1. Flexibility—Range of Motion

The average distance reached in the sit-and-reach test was 26 cm, with considerable variability in flexibility among participants, ranging from 11 cm (minimum score) to 43 cm (maximum score). The vast majority of the participants showed either a medium level (43% of the sample; 14–17 cm) or good level (51% of the sample; above 18 cm) in the sit-and-reach score, whereas a small percentage (6%) showed a low level (below 13 cm) of sit-and-reach score, according to the ACSM [48] and Golding [56] classifications. Concerning the back scratch test, the mean values were 2.46 cm for the right hand and 5.95 cm for the left hand, with great variation among participants, ranging from −12.50 to +15 cm and from −7 to +21.5, respectively. It should be mentioned that a significant proportion of the participants showed low (30%) or medium (20%) levels in the back scratch test score of the right hand, while in the left hand, the vast majority of the participants (68%) showed good score in the back scratch test and only a small percentage (12%) showed a low score, according to the Corbin et al. [55] classification. Furthermore, the paired t-test indicated a statistically significant difference in the back scratch test (t_99_ = −5.348 and *p* = 0.000), where the left arm showed a higher value than the right arm.

#### 3.2.2. Balance

Regarding the single-limb stance test, the results showed a) considerable variability in balance performance, ranging from 1.03 to 141.25 sec for the right leg and from 1.22 to 137.06 sec for the left leg, and b) a statistically significant difference in static balance between the two legs (t_99_ = 2.55 and *p* = 0.012), where the static balance of the right leg was higher compared to the left leg. It should be also mentioned that in the right leg, 23% of the participants showed low levels of static balance, 19% medium levels, and 58% good levels, whereas in the left leg, 30% of the participants showed low levels of static balance, 26% medium levels, and 44% good levels, according to the Rinne et al. [51] classification. In the TUG (time up-and-go) test, the mean and median values are both 6 sec, indicating a consistent performance across all participants. The low standard deviation (0.70) indicates minimal variability in the time taken to complete the task.

#### 3.2.3. Strength—Aerobic Capacity

The participants’ mean performance on the upper body endurance strength test was six push-ups, with a wide range of strength levels among the participants, ranging from zero to nineteen push-ups. In the push-up test, the vast majority of the participants (60%) showed low performance (<6 push-ups), 32% showed medium performance (7–13 push-ups) and 8% showed good performance (≥15 push-ups), according to the ACSM classification [48]. Moreover, the mean resting heart rate was 72.26 beats/min, ranging from 47 beats/min to 91 beats/min, while the mean heart rate 1 min after the test was 106.33 beats/min, ranging from 72 beats/min to 144 beats/min. The descriptive statistics of functional capacity and physical fitness indices are analytically presented in Table 6.

### 3.3. Lifestyle Behaviors

#### 3.3.1. Smoking

According to the results of the study, 64% of the participants stated that they are not smokers, and the rest 36% have been smoking on average for 17.64 ± 10.79 years (ranging from 1 to 39 years). Regarding the quantity of smoking per day, 16% of the participants smoke less than a half pack of cigarettes/day, 12% smoke half to a whole pack per day, and 8% smoke one to one-and-a-half packs per day.

#### 3.3.2. Alcohol Consumption

**Figure 2:**
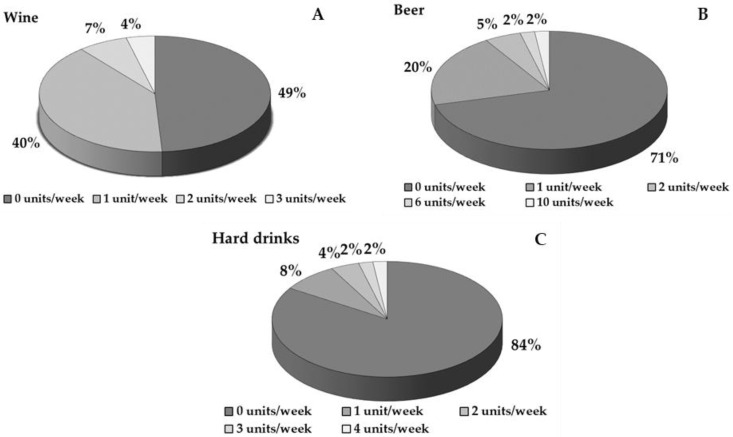
Incidence of alcoholic beverages consumption per week for wine (**A**), beer (**B**), and hard drinks (**C**).

In total, 62% of the participants drink alcoholic beverages and the other 38% do not. It should be mentioned that the vast majority of the participants prefer wine as an alcoholic beverage, while 29% of the participants consume beer each week. On the other hand, only 16% of the participants consume hard drinks each week. The incidence of alcohol consumption is analytically presented in Figure 2.

#### 3.3.3. Caffeinated Beverage Consumption

The majority of the participants (92%) answered that they consume caffeinated beverages each week, whereas the other 8% do not consume them. It should be also mentioned that all the participants that consume caffeinated beverages each week drink coffee (where 78% consume 7–14 coffees/week), while 19% consume tea (1–8 cups of tea/week) and 24% consume soft drinks each week. The incidence of caffeinated beverage (coffee, tea, soft drinks) consumption is analytically presented in Table 7.

#### 3.3.4. Screen Time Levels

**Figure 3:**
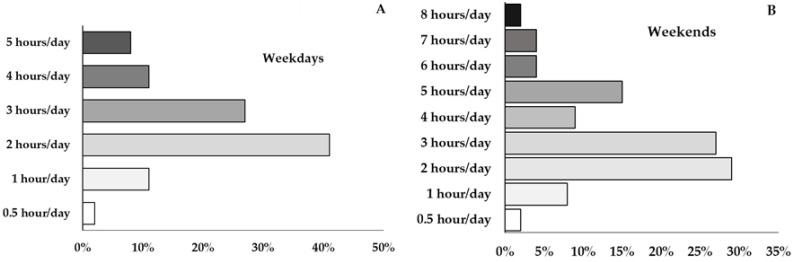
Incidence of screen time levels on weekdays (**A**) and weekends (**B**) in female healthcare professionals (*n* = 100).

As regards time spent on sedentary screen activities (tablet, video, mobile phone, TV shows), the results showed that 44% of the participants showed increased screen time levels (≥3 h/day) above the recommended limits during weekdays, whereas for the remaining 56%, the screen time levels were within permissible limits. On the other hand, on weekends a great percentage of participants (61%) reported increased screen time levels (ranging from 3 to 8 h/day), while for the remaining 39% of the participants the screen time levels ranged within permissible limits from 0.5 to 2 h/day. The incidence of screen time levels on weekdays and weekends is analytically presented in Figure 3.

#### 3.3.5. Physical Activity Levels

Concerning the question “Do you participate in physical activities?”, 33% of respondents (*n* = 33) reported participating in physical activities, while 67% (*n* = 67) did not. Among those who reported participation, the frequency varied as follows: 10% participated once a week, 7% twice a week, 8% participated three times a week and 8% participated 4–6 times per week. The duration of physical activities per day for the vast majority of the participants (31%) was 1 h a day, while a small percentage (2%) reported spending ½ hour per day on physical activities.

## 4. Discussion

The main purpose of this study was to measure and evaluate various health, functional capacity, and physical fitness indices as well as lifestyle behaviors in Greek female healthcare professionals, where the scientific literature is limited. This study showed a high prevalence of overweight and obesity, high incidence of musculoskeletal pains, and increased levels of blood pressure and body circumference in a significant proportion of the sample. The present study also demonstrated decreased levels of functional capacity, physical fitness, and physical activity levels, as well as increased levels of viewing habits and a significant prevalence of smoking. The results of the present study provide valuable information regarding the wellness profile of Greek female healthcare professionals. This information can be used for the effective and safe design and implementation of workplace wellness interventions for this specific population, aiming to improve workers’ health and quality of life as well as work ability and productivity.

### 4.1. Musculoskeletal Pains

Our study found that the majority of healthcare employees (96%) had musculoskeletal pains in various body parts, especially in the lower back (77%), upper back (71%), neck (56%), knee (47%), shoulder (44%), and hip (41%). Our findings are consistent with previous studies that were carried out among the Greek healthcare working population, indicating a high incidence of musculoskeletal complaints [27,28,29,32]. In more detail, Alexopoulos et al. [27] examined the prevalence of musculoskeletal complaints in three body parts (back, neck, and shoulder), indicating that the lower back was the most pained body part among nursing personnel, reported by 75% of the participants, followed by the neck (47%), and the shoulder (37%). Alexopoulos et al. [27] also showed high musculoskeletal co-morbidity, since 75% of the participants indicated pain in more than one body part. The findings of Alexopoulos et al. [27] regarding musculoskeletal co-morbidity have been strengthened by the results of the present study, where the vast majority of the participants (89%) showed pain in more than one body part. In the same context, Bakola et al. [29] reported a high incidence of musculoskeletal problems in Greek nurses, especially in the lumbar spine (54.4%), neck (47.7%), and shoulder (45.5%), followed by lower percentages of pain in the hip, knee, elbow, and ankle, as well as high musculoskeletal co-morbidity, since 74.9% of the participants indicated pain in two or more body parts. Furthermore, Passali et al. [32] reported a high prevalence of musculoskeletal disorders (at the waist, neck, and back) in Greek nurses (98% of the participants), indicating that female nurses, as well as nurses with greater work experience, show higher risk of musculoskeletal disorders than males and younger nurses with less work experience. The results of the present study are also in accordance with previous studies in other countries that have reported a high prevalence of musculoskeletal complaints [57,58,59,60,61,62], with some differences in the amount of prevalence. A previous study [28], which compared the musculoskeletal complaints between Greek and Dutch nurses, demonstrated more back and neck complaints in the preceding 12 months for Greek nurses (back: 75% and neck: 47%) compared to Dutch nurses (back: 62% and neck: 39%), while chronicity and sickness absence of these complaints did not differ between Greek and Dutch nurses. Finally, in our study, a significant proportion of the participants (56%), although not absent from work, mentioned that their musculoskeletal pains contributed to the deceleration of daily activities at the workplace, with all the possible effects on the reduction in productivity as well as the quality of provided patient care. Other studies have found significant percentages of work absenteeism due to musculoskeletal pain in healthcare professionals [27,28,63].

### 4.2. Other Health Indices

Our research findings demonstrate that the obesity prevalence among Greek female healthcare professionals is considered high, since 39% of the participants were overweight and 28% were obese. Our results are in agreement with those of previous studies, which have found high levels of obesity and/or overweight among health workers all over the world [30,31,34,64]. Specifically, Elabd et al. reported a high prevalence of obesity (36.8% for females and 38.7% for males) among hospital employees in Saudi Arabia [30], while Kyle et al. found that about 25% of nurses and 14% of other healthcare professionals in UK were obese [31]. Additionally, a previous study has found an obesity prevalence of 47.3% in healthcare employees from the United Arab Emirates [64]. In Greece, the situation is particularly worrying, as in recent years there has been a rapid increase in obesity levels both in the general population and in healthcare workers. A previous study from Tountas et al. showed that 25% of Greek female hospital employees of different specialties were overweight and 9% were obese [34]. By comparing the obesity rates of this study [34] with those of the present study, we can see the rapid change in obesity prevalence in Greek female healthcare employees. Aside from the high prevalence of overweight and obesity, in our study, we found that a significant proportion of Greek healthcare employees have systolic blood pressure of more than 130 mmHg (25% of the participants) and diastolic blood pressure ≥ 85 mmHg (20% of the participants), as well as a waist circumference of ≥88 cm and a waist-to-hip ratio of ≥0.86 (45–49% of the participants). Our findings are in line with previous studies which have also reported increased levels of blood pressure and body circumference in health workers [33]. The high levels of overweight and obesity in conjunction with the increased levels of blood pressure and body circumference observed in this study and previous studies may be characterized as significant predisposing risk factors for the appearance of health-related problems such as cardiovascular diseases, hypertension, metabolic syndrome, etc. [65].

### 4.3. Functional Capacity—Physical Fitness and Lifestyle Behaviors

In the present study, we found that a significant proportion of Greek female healthcare workers showed (a) low–medium levels of functional capacity and physical fitness levels (particularly in the push-up test, time up-and-go test, and aerobic step test) and (b) significant differences in flexibility (sit-and-reach test) and static balance (single-limb stance test) between the two limbs (right and left). The low–medium levels of functional and physical fitness levels, as well as the differences between limbs observed in this study, may be attributed either to the absence of physical activity or to the inadequate levels of physical activity. Indeed, the vast majority of the sample in the present study (67%) did not participate in physical activities, while, among those who reported participation in physical activities, only 16% followed the guidelines of the ACSM [65] for systematic participation (at least three times per week). In the scientific literature, the limited studies that have measured and evaluated functional capacity and physical fitness indicators in healthcare workers or nursing students also reported low–below average levels in different physical abilities [36,66,67,68]. The results of this and previous studies reinforce the recommendations of the CDC and World Health Organization (WHO) for the regular participation of healthcare workers in physical activities to improve functional capacity and physical fitness levels. The reduced levels of physical activity, as well as the increased sedentary behaviors, may negatively affect individuals’ health and quality of life [65]. In the present study, we found increased screen time levels on weekdays and weekends for a significant proportion of female healthcare workers, showcasing the impact of digital engagement on daily routines. Our findings corroborate previous research that documented increased sedentary habits among healthcare professionals [45,46].

Concerning other lifestyle behaviors, our study demonstrated that 36% of Greek female healthcare employees are smokers. This percentage is similar to recent studies in Greek healthcare populations [69] but lower than that observed in older studies [39]. According to the survey by the European Observatory on Health Systems and Policies [70], smoking rates among Greek males and females have decreased but continue to be high compared to other EU countries. Furthermore, it should be also mentioned that smoking prevalence in the Greek healthcare population, although decreased during recent years [70], continues to be higher than that observed in the Greek general population as well as in the healthcare population from other countries. Additionally, in the present study, a substantial proportion (62%) reported consuming alcoholic beverages, with varying frequencies across different types of drinks, but no alcohol abuse was found, as mentioned in other previous studies. Finally, caffeinated beverage consumption was prevalent in the present study, with 92% affirming consumption, and coffee being the most common choice (the vast majority consume 1–2 coffees per day). Similarly, a previous study on nurses from three different countries (Italy, Korea, and the USA) showed consumption of at least one caffeinated beverage per day [47].

The present study also has some limitations that could affect the collection of the results and therefore their generalization. The present study measured and examined various health, functional capacity, and physical fitness indices, as well as lifestyle behaviors in Greek female healthcare professionals, but did not examine the association/interaction between the aforementioned indicators. Future studies could examine the associations between health indices, functional capacity, physical fitness indices, lifestyle behaviors, and work ability to draw more effective and safer conclusions regarding their interaction. Another important limitation of this study is the diversity of the sample, including participants from different specialties (e.g., doctors, nurses, ergotherapists, physiotherapists, medical laboratory technicians), which could affect our findings. Unfortunately, in the present study, we did not have the appropriate participants per specialty to present the results separately and examine the differences in wellness profiles between specialties. Future studies (with a greater sample size per specialty) could examine and compare possible differences in wellness profiles between healthcare professionals of different specialties. Finally, the participants’ characteristics (e.g., sex, age), profession, and staff category, as well as the working characteristics (e.g., work experience in years) of the participants are important limitations that could affect the generalization of our findings. Future studies should examine and create a comprehensive wellness profile in other professions and staff specialties, in younger employees, and in employees who have different working characteristics.

## 5. Conclusions

In conclusion, our study reveals that musculoskeletal pains are prevalent among healthcare professionals, impacting daily working activities despite minimal work absenteeism. Additionally, the high prevalence of overweight and obesity and the increased levels of blood pressure and body circumference observed in a significant proportion of the sample emphasize the need for tailored wellness initiatives for the aforementioned health indicators. Regular exercise and specific nutrition workplace interventions could be efficient strategies for the improvement of the aforementioned health parameters (reduction in body fat, blood pressure, and body circumference), as well as for the prevention and rehabilitation of chronic diseases (i.e., obesity, cardiovascular diseases). Furthermore, the high incidence of musculoskeletal pains observed in this and previous studies strengthen the importance of designing and implementing specific interventions—exercise interventions aiming to improve flexibility and muscle strength, as well as ergonomic interventions for the prevention and rehabilitation of musculoskeletal pains. On the other hand, the decreased levels of functional capacity, physical fitness, and physical activity levels and the increased levels of viewing habits underscore the importance of a holistic physical activity and exercise workplace approach for this population (combined exercise programs to improve all functional capacity and physical fitness indices, tips to increase physical activity at work using physical activity breaks, etc.). Finally, the significant prevalence of smoking in female healthcare workers, as well as the alcohol and caffeine consumption patterns, highlight lifestyle influences on healthcare professionals’ well-being. Addressing all these factors mentioned above is crucial for supporting the health and performance of healthcare workers, ultimately ensuring high-quality patient care.

## Figures and Tables

**Table 1:** Demographic, anthropometric, and working characteristics of the female healthcare professionals (*n* = 100).

Variables	Mean	Std. Deviation
Demographic and anthropometric characteristics		
Age (years)	45.53	2.00
Body height (m)	1.65	0.05
Body mass (kg)	74.38	11.24
Body mass index (kg/m^2^)	27.37	4.56
Working characteristics		
Working experience (years)	10.76	5.02
Working hours/day (hours)	9.50	1.00

**Table 2:** Health, functional capacity, physical fitness, and lifestyle behavior measurements.

Measured Index	Test/Protocol	Equipment/Questionnaire
Health indices		
Musculoskeletal pains	The presence of musculoskeletal pains (duration and intensity) during the last month in 9 body parts (neck, shoulders, elbows, wrists/hands, upper back, lower back, hip, knees, foot/ankle) were evaluated. The days of work absenteeism and the negative impact of musculoskeletal pains on participants’ daily activities were also assessed during the last month.	Nordic Musculoskeletal Questionnaire [53].
Body mass— Body height/ Body mass index	The body mass (to the nearest 0.5 kg) and the body height (to the nearest 0.1 cm) were measured, and then the body mass index was calculated, according to the recommendations of ACSM [48].	Digital scale (Tanita BC-587). Measuring tape fixed to a wall.
Body fat	The percentage of body fat (%BF) was assessed, according to the recommendations of ACSM [48].	Digital scale (Tanita BC-587).
Body circumference	The waist and hip circumferences were measured, according to the recommendations of ACSM [48], and thereafter the waist-to-hip ratio was calculated.	Ergonomic circumference measuring tape (Seca 201).
Blood pressure	The blood pressure (systolic and diastolic) was measured, while participants were seated and rested for 10 min before the measurement.	Electronic blood pressure monitor (OMRON MIT Elite plus).
Respiratory function (spirometry)	The respiratory function (forced vital capacity-FVC, forced expiratory volume in 1 s-FEV_1_) was measured using spirometry, according to the recommendations of the American Thoracic Society [54].	Portable spirometer (MIR Spirobank).
Functional capacity and physical fitness indices		
Flexibility—Range of motion	Lower body flexibility was measured using the sit-and-reach test [48] and upper body range of motion was assessed on both hands using the back scratch test [55]. Three maximal trials (10 s rest/trial) were performed at each test and the best score (in cm) was considered for analysis.	Flex-Tester box (Novel Products Inc, Rockton, IL, USA). Measuring tape.
Balance	The single-limb stance test (with eyes opened) was used to assess static balance on both legs [51]. Three trials were performed on each leg and the average (time in s) was considered for analysis. The timed up-and-go test was used to measure dynamic balance [52]. Three maximal trials (rest: 30 s/trial) were performed and the best time (in s) was considered for analysis.	Stable chair (without wheels and armrests). Athletic cone. Stopwatch.
Strength	The muscular endurance of the chest and triceps muscles was assessed, using the modified knee push-up test according to the ACSM’s instructions [48].	Gymnastic mat.
Aerobic capacity	The participants’ aerobic capacity was measured using the YMCA 3 min step test, which was performed following the metronome cadence (96 beats per minute; 4 steps per cycle). The participants’ HR was measured before the test as well as 1 min following the termination of the step test [56].	Platform/step (height: 30 cm). Electronic metronome. Stopwatch. Heart rate monitor.
Lifestyle behaviors		
➢ Smoking: Smoking behaviors were examined using four questions. 1. Do you smoke? 2. How many years have you been smoking? 3. How much do you smoke per day? 4. Did you smoke in the past and if so, when did you quit? ➢ Alcohol consumption: The total weekly consumption of alcoholic beverages (a. wine, b. beer, and c. hard drinks) was measured using four questions. 1. Do you drink alcoholic beverages? 2. How many units of wine do you consume per week? 3. How many units of beer do you consume per week? 4. How many units of hard drinks do you consume per week? ➢ Caffeinated beverages consumption: The total weekly consumption of caffeinated beverages (a. coffee, b. tea, and c. soft drinks) was measured using four questions. 1. Do you drink caffeinated beverages? 2. How many units of coffee do you consume per week? 3. How many units of tea do you consume per week? 4. How many units of soft drinks do you consume per week? ➢ Physical activity levels: The weekly frequency and duration of participation in physical activities were measured using three questions. 1. Do you participate in physical activities? 2. How many days per week do you participate in physical activities? 3. How many hours per day do you participate in physical activities? ➢ Screen time levels: The total screen time levels during participants’ free time were examined using two questions. 1. On weekdays (in your free time), how much time do you watch TV, play with your tablet, PC, videogames, or mobile phone per day? 2. On weekends (in your free time), how much time do you watch TV, play with your tablet, PC, videogames, or mobile phone per day?		ACSM Health Status and Health History Questionnaire [48].

**Table 3:** Duration (number of days) of pain during the previous month in the nine body parts.

Body Parts	Number of Days				
	0 Days	1–7 Days	8–14 Days	15–21 Days	>22 Days—Every Day
Neck	44%	31%	5%	5%	15%
Shoulder	56%	25%	3%	3%	13%
Upper back	29%	33%	6%	6%	26%
Elbow	80%	18%	0%	1%	1%
Wrist/hands	64%	19%	5%	6%	6%
Lower back	23%	43%	5%	6%	23%
Hip	59%	24%	3%	3%	11%
Knee	53%	25%	6%	6%	10%
Foot/ankle	77%	11%	4%	4%	4%

**Table 4:** Intensity of pain (0–10 scale) during the previous month in the nine body parts.

Body Parts	Score on 10-Point Scale										
	0	1	2	3	4	5	6	7	8	9	10
Neck	44%	0%	6%	15%	7%	6%	6%	3%	4%	5%	4%
Shoulder	56%	3%	5%	10%	6%	6%	2%	0%	5%	4%	3%
Upper back	29%	4%	6%	14%	10%	7%	8%	4%	5%	4%	9%
Elbow	80%	6%	4%	2%	2%	3%	0%	2%	1%	0%	0%
Wrist/hands	64%	8%	8%	3%	2%	2%	4%	4%	2%	2%	1%
Lower back	23%	2%	11%	10%	9%	7%	16%	5%	13%	4%	0%
Hip	59%	9%	3%	3%	5%	8%	0%	4%	2%	4%	3%
Knee	53%	8%	2%	10%	7%	4%	4%	4%	4%	2%	2%
Foot/ankle	77%	3%	10%	2%	2%	0%	2%	0%	0%	2%	2%

**Table 5:** Descriptive statistics for body composition and circumference, blood pressure, and respiratory function in female healthcare professionals (*n* = 100).

Variables	Mean	Median	SD	Minimum	Maximum
BMI (kg/m^2^)	27.37	26.88	4.56	18.50	37.96
Body fat (%)	35.92	37.05	6.73	18.40	48.90
Waist circumference	88.30	86.50	12.22	65.50	122.00
Hip circumference	101.70	100.50	11.02	74.00	132.00
Waist-to-hip ratio	0.87	0.84	0.10	0.73	1.19
Systolic BP (mm/Hg)	122.60	120.00	15.33	101.00	171.00
Diastolic BP (mm/Hg)	75.60	75.00	11.45	50.00	113.00
FVC (L/min)	3.47	3.53	0.47	2.22	4.38
FEV_1_ (L/min)	2.97	2.99	0.38	1.91	3.75

SD: standard deviation, BMI: body mass index, BP: blood pressure, FVC: forced vital capacity, FEV_1_: forced expiratory volume in 1 s.

**Table 6:** Descriptive statistics for functional capacity and physical fitness indices in female healthcare professionals (*n* = 100).

Tests	Mean	Median	SD	Minimum	Maximum
Flexibility—Range of motion					
Sit-and-reach (cm)	26.00	24.00	7.61	11.00	43.00
Back scratch test—Right hand (cm)	2.46	2.75	6.89	−12.50	15.00
Back scratch test—Left hand (cm)	5.95	6.00	6.75	−7.00	21.50
Balance					
Static balance test—Right leg (s)	51.70	45.53	34.24	1.03	141.25
Static balance test—Left leg (s)	44.85	32.67	35.02	1.22	137.06
TUG test (s)	6.00	6.00	0.70	5.00	8.00
Strength—Aerobic capacity					
Push-up test (repetitions)	6.00	4.00	4.92	0.00	19.00
HR resting (beats/min)	72.26	74.00	8.23	47.00	91.00
HR 1’after test (beats/min)	106.33	106.50	15.62	72.00	144.00

SD: standard deviation, TUG test: time up-and-go test, HR: heart rate.

**Table 7:** Incidence of caffeinated beverage consumption per week in female healthcare professionals (*n* = 100).

Coffee consumption per week (number of coffees)												
0 coffees	4 coffees		7 coffees	9 coffees	10 coffees		12 coffees	14 coffees	15 coffees	20 coffees		21 coffees
8%	2%		20%	2%	18%		4%	30%	10%	4%		2%
Tea consumption per week (number of cups)												
0 cups		1 cup		3 cups		4 cups		5 cups	7 cups		8 cups	
81%		4%		2%		4%		4%	3%		2%	
Soft drink consumption per week (number of soft drinks)												
0 soft drinks		1 soft drink		2 soft drinks		4 soft drinks		7 soft drinks	14 soft drinks		40 soft drinks	
76%		10%		6%		2%		2%	2%		2%	

## Data Availability

Data are unavailable due to privacy or ethical restrictions.

## Supplementary Materials

The following supporting information can be downloaded at https://www.mdpi.com/article/10.3390/healthcare13040383/s1, Table S1: Normal distribution results and skewness—kurtosis values for health indices, functional capacity, and physical fitness.
