# Supplementary material for: Measurement and Evaluation of Health, Functional Capacity, Physical Fitness, and Daily Habits of Greek Female Healthcare Professionals Working in a Hospital Environment
Source: Healthcare (Basel). 2025 Feb 11;13(4):383. doi: 10.3390/healthcare13040383 (PMC11855844; doi:10.3390/healthcare13040383)
Supplement: Supplementary file 1 [file healthcare-13-00383-s001.zip › healthcare-3381666-supplementary.pdf]

Table S1. Normal distribution results and skewness - kurtosis values for health indices, functional capacity and physical fitness.

**Variables**	Normality test (Kolmogorov-Smirnov)		Skewness		Kurtosis	
	Statistic	Sig.	Statistic	Std. Error	Statistic	Std. Error
Health indices						
BMI	0.085	0.162	0.637	0.246	-0.270	0.488
Body Fat	0.087	0.164	-0.399	0.246	0.026	0.488
Waist circumference	0.088	0.160	0.600	0.246	-0.033	0.488
Hip circumference	0.089	0.157	0.309	0.246	0.411	0.488
Systolic BP	0.090	0.158	0.601	0.246	0.577	0.488
Diastolic BP	0.079	0.169	0.675	0.246	1.367	0.488
FVC	0.091	0.157	-0.446	0.246	-0.083	0.488
FEV_1	0.059	0.200	-0.219	0.246	0.145	0.488
Functional capacity						
Flexibility						
Sit and Reach	0.089	0.075	0.535	0.246	-0.212	0.488
Back scratch test - Right hand	0.095	0.072	0.052	0.246	-0.617	0.488
Back scratch test - Left hand	0.060	0.200	0.175	0.246	-0.512	0.488
Balance						
Static balance test - Right leg	0.085	0.081	0.625	0.246	0.035	0.488
Static balance test - Left leg	0.099	0.061	0.780	0.246	0.548	0.488
TUG test	0.091	0.072	0.610	0.246	-0.087	0.488
Physical fitness						
Strength						
Push-up test	0.100	0.062	0.580	0.246	-0.704	0.488
Aerobic capacity						
HR resting	0.095	0.093	-0.624	0.246	0.890	0.488
HR 1΄after test	0.079	0.162	0.066	0.246	-0.257	0.488

BMI: body mass index, BP: blood pressure, FVC: forced vital capacity, FEV_1: forced expiratory volume in 1 s. TUG test: time up-and-go test, HR: heart rate.
